# Modern Approaches and Innovations on Methods and Imaging Protocols of the Maxillofacial District

**DOI:** 10.3390/mps5010002

**Published:** 2021-12-27

**Authors:** Rodolfo Reda, Maurilio D’Angelo, Alessio Zanza, Dario Di Nardo, Luca Testarelli

**Affiliations:** Department of Oral and Maxillofacial Sciences, Sapienza University of Rome, 00161 Rome, Italy; rodolfo.reda@uniroma1.it (R.R.); maurilio.dangelo@uniroma1.it (M.D.); dario.dinardo@uniroma1.it (D.D.N.); luca.testarelli@uniroma1.it (L.T.)

In recent years, improvements in imaging techniques have profoundly changed the diagnosis of pathologies of the maxillofacial district. Three-dimensional radiographic diagnostic exams, with a lower dose of ionizing radiation and the same, or a higher, resolution, are frequently applied to dentistry and the maxillofacial district for the diagnosis and treatment of pathologies and conditions that, until a few years ago, required several radiographic examinations [1,2,3,4]. 

Cone Beam computed tomography (CBCT) represents the most widespread and most-used 3D exam in dentistry. This diffusion is allowed because the equipment is the least voluminous, allows extremely reduced, high-quality acquisition times compared to other tests, and has a lower cost than other diagnostics equipment, such as Magnetic Resonance Imaging (MRI) [4,5,6].

The ability to modify the FoV while maintaining a very high image quality and the functionality of various image management and re-processing software allow for its use in dentistry, ranging from use in the smallest FoVs for a few dental elements in endodontics to larger FoVs, for example, in orthodontics [4,7]. In endodontics, a careful anatomical evaluation is increasingly important to complete the diagnosis and improve the prognosis of endodontic treatments, avoiding iatrogenic errors such as fractures in rotating instruments and perforations [8,9,10,11,12].

Moreover, this also allows for the study of the temporomandibular joint in gnathology, offering most of the indications that are useful to the clinician when completing the treatment plan, assisted by MRI [5]. Therefore, considering the possible widespread application of this exam, it is necessary to underline how many guidelines on the prescription of first- and second-level exams will require updates, thus, thanks to the new image processing and calculation software, greatly reducing the number of tests that are prescribed to confirm the diagnosis [13].

The main and most relevant dilemma in the use of this technique is represented by the presence of ionizing radiation, the importance of which is often underestimated in the prescription of this kind of diagnostic exam [5]. In this regard, an increasing amount of attention is being paid to radiation-free imaging techniques, from MRI to imaging techniques that use ultrasound. The use of both in clinical dentistry is increasingly being investigated, and the main difference between them is represented by the learning curve necessary to perform a good ultrasound examination; it is greater than that needed for MRI, which is a more complex examination, due to the long times needed for the acquisition of high-resolution data [5,14,15,16].

Considering the ultrasounds and their physical characteristics, their application in dentistry, considering the superficiality of the structures to be studied, is extremely valid. Vascular lesions of the tissues of the oral cavity, of the oral tissues themselves, or of the more superficial bone and alterations, with the appropriate probes, seem to be simple to apply, free of ionizing radiation, minimally invasive, and easily available in the office for constant use during daily clinical practice [14,15].

Recent evidence makes it possible to consider MRI as a complete dental diagnostic examination, which allows for both an investigation of the anatomy of the soft tissues at certain frequencies and the volumes and bone density [16]. Furthermore, recently published evidence shows that MRI is currently comparable, in the planning of implant surgery, to the static or dynamic guided surgery planned using CBCT [5,17]. Furthermore, improvements in the evaluation of the anatomy of the endodontic system and in the guided surgery, with templates produced exclusively based on this diagnostic examination, were important [10,11,18,19]. This represents a major step towards radiation-free diagnostics, which are increasingly innovative and protective of the patient.

Unlike resonance, ultrasound allows for a more specific evaluation of a superficial mucous or bone site, but allows for the possibility of modifying the parameters to increase the definition of the collected images or the depth of the vision. With specific parameters, this examination also allows for the evaluation of dental hard tissues, which has not been explored with this method to date. It is non-invasive, does not cause biological damage, and avoids the use of intraoral periapical or bite-wing radiographs [14,15]. 

Further in-depth studies and in vitro studies will be required to ascertain the effectiveness and innovation brought about by the application of these techniques for a use that is not practiced today, and, subsequently, the instruments for oral use will have to be implemented, especially the probes for diagnostic examinations with ultrasound.

The margins of success are wide, guided by the non-invasiveness of these procedures and the absence of biological damage caused by ionizing radiation.
